# Zener-like electrical transport in polyaniline–graphene oxide nanocomposites

**DOI:** 10.1039/c9ra07267e

**Published:** 2020-01-29

**Authors:** Animesh Kr. Dey, Gaurav Kumar, Pradip K. Maji, R. K. Chakrabarty, U. N. Nandi

**Affiliations:** Department of Physics, Scottish Church College 1 & 3 Urquhart Square Kolkata 700 006 India un_nandi@yahoo.co.in; Department of Polymer and Process Engineering, Indian Institute of Technology Roorkee India; Department of Physics, Government College of Ceramic Engineering and Technology Kolkata 700 010 India

## Abstract

The present study includes the fabrication and characterization and an investigation of the electrical transport properties of nanocomposites of n-PANI and graphene oxide (GO). The samples were prepared by loading different weight percentages *D* of GO during the chemical oxidative *in situ* polymerization of aniline monomers. Structural characterization by XRD, FTIR, FESEM, *etc.* confirmed that the nanocomposites exhibited superior morphology and thermal stability. The transport properties were studied by measuring the variation of conductivity with temperature *T*, *V*–*I* characteristics and the fundamental response *V*_f_ at different temperatures *T*. The dc conductance *Σ* showed a transition from insulator type behavior to weakly temperature dependent behavior at temperature *T*_D_, which decreased with increasing *D*. The *V*–*I* characteristics were generally nonlinear and the nonlinearity increased with decreasing temperature. Moreover, at temperatures *T* ≥ *T*_D_, the characteristics showed saturation of voltage for higher values of current, similar to Zener diodes. At lower temperatures (*T* ≤ *T*_D_), a voltage maximum occurred, similar to thyristors. This behavior leads to the possibility of fabricating devices containing these nanocomposites. We have tried to analyze these results using the framework of scaling theory and the concept of inter-chain hopping conduction and tunneling between conducting grains separated by insulating regimes in the nanocomposite.

## Abstract 2

The present study includes the fabrication and characterization and an investigation of the electrical transport properties of nanocomposites of n-PANI and graphene oxide (GO).

## Graphical abstract

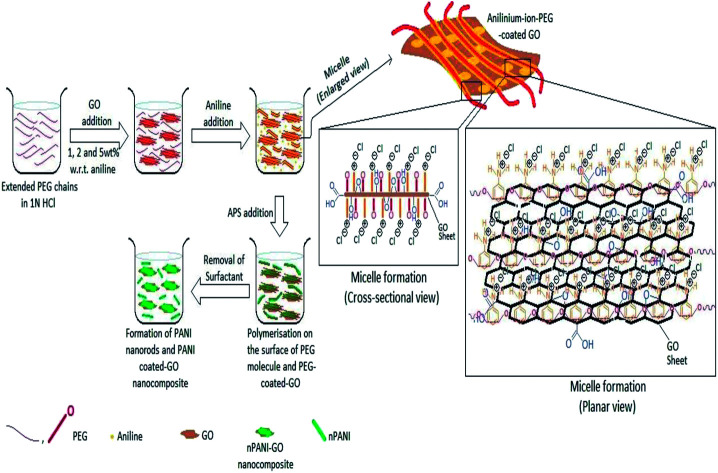

## Introduction

Polyaniline (PANI) is one of the most studied organic polymers in materials science.^1,2^ PANI exhibits exceptional physico-chemical properties such as flexibility, solution processibility and tunable conductivity on undergoing reversible doping processes. Greater chemical and environmental stability, ease of synthesis and the large-scale availability of low cost monomers^3^ make this polymer a good candidate for the development of functional carbon based polymer composites and the fabrication of numerous technological devices such as supercapacitors,^4–6^ sensors,^7,8^ electronic devices,^9^ batteries^10,11^ and light emitting diodes.^12^ This polymer is available in three distinct oxidation states:^13^ the fully reduced leucoemeraldine base (LB) (–(C_24_H_20_N_4_)_*x*_–), the half-oxidized emeraldine base (EB) (–(C_24_H_18_N_4_)_*x*_–) and the fully oxidized pernigraniline base (PNB) (–(C_24_H_16_N_4_)_*x*_–). Both LB and EB are insulators with a large extrinsic gap *E*_g_ ∼ 3.6 eV whereas PNB possesses an energy gap *E*_g_ ∼ 1.4 eV and shows conducting properties due to electron–phonon interactions.^14^ The conducting emeraldine salt (ES) form of the polymer is achieved upon protonation of EB by exposure to protic acids or upon oxidative doping of LB. This ES state is composed of two benzoid units and one quinoid unit that alternate and is regarded as the most useful form of polyaniline with semiconducting properties. This intrinsically conducting property^1^ of the ES state of the polymer attracts researchers to explore its outstanding electrical, magnetic, electro-chemical, thermo-electrical, and optical properties.

In order to achieve superior electrical, thermal and mechanical properties to the corresponding component materials, various nanofillers such as camphor sulfonic acid,^15^ graphene,^16–18^ carbon nanotubes,^19,20^ graphene oxide,^21–26^ and reduced graphene oxide^27–29^ have been added to PANI to fabricate innovative polyaniline nanocomposites of significant technological and scientific importance. Out of these carbon based materials, graphene oxide (GO) is extensively used as a filler in PANI because of its higher chemical stability, the easy availability of a low cost precursor (natural graphite), and the greater feasibility of large-scale production. Further, GO has structural advantages in which the edges are decorated with tunable polar oxygen-containing hydrophilic functional groups such as hydroxyl groups, carboxyl groups and epoxides.^22^ These functional groups exhibit strong interfacial interactions with polar molecules and polymers resulting in intercalated or exfoliated GO-based polymer nanocomposites.^30–32^ Moreover, the thermal stability of these nanocomposites is enhanced to a greater extent due to entrapment of PANI by GO^33^ and makes these composites suitable to be used in devices such as sensors, radar absorbing systems and energy storage elements^16,34,35^ for applications in the aerospace industry.^21^ These nPANI–GO nanocomposites are also applied as supercapacitor materials^23,36^ and have achieved a very high value of specific capacitance of the order of 531 F g^−1^ at 0.2 A g^−1^ in aqueous electrolyte,^16^ an electrochemical capacitance of 233 F g^−1^ for a graphene–PANI composite,^36^ and a capacitance of 425 F g^−1^ at a current density of 0.2 A g^−1^ with a supercritical carbon dioxide (SC CO_2_) support and an aniline concentration of 0.1 mol L^−1^.^37^ Using this nanocomposite, Xia *et al.*^38^ obtained an EDL (electric double-layer) capacitance of nearly 21 μF cm^−2^ and determined the quantum capacitance of single layer and double-layer graphene.

Except for a few studies on the electrical transport properties of such nPANI–GO nanocomposites,^13,15,26,39–45^ investigations so far have been mainly focused on the synthesis, characterization and practical applications. Systematic studies of the electrical conductivity as a function of temperature and electric field both in the ohmic and non-ohmic regions are rather scant in the literature and require proper attention for better understanding of the electrical transport properties in the non-ohmic region to examine the possibility of technological applications. In this paper, we report experimental results on the electrical conductance *Σ* of nPANI–GO nanocomposites as a function of voltage *V* and temperature *T* for different weight percentages *D* of GO. The main objectives of this article are the following: (i) characterization of the morphological structure and chemical properties of nPANI–graphene oxide composites using XRD, FTIR, FESEM and UV-visible spectroscopy, (ii) determination of the conduction mechanism considering the potential interactions between nPANI polar groups and various oxygen-containing functional groups, (iii) investigation of the electrical transport properties by measuring *V*–*I* characteristics and the fundamental response *V*_f_ as a function of voltage and temperature in both the ohmic and non-ohmic regions, and (iv) analysis of the experimental results using scaling formalism in terms of the onset voltage *V*_0_(*T*) and the nonlinearity exponent *x*_T_ to examine the possible scope of technological applications. The paper is organized in the following way: the materials and methods for the preparation and characterization of the nPANI–GO composites and the procedure for measuring the electrical transport properties are described first. Experimental results on the variation of *R*–*T*, *V*–*I* and the fundamental response *V*_f_ are then presented and analyzed using scaling formalism. The salient features of the non-ohmic electrical transport properties including the Zener-like features at different temperatures are discussed, highlighting possible applications. Finally, the results are summarized in the Conclusion section.

## Materials and methods

### Synthesis and purification of GO

GO was prepared by the oxidation of natural graphite powder according to Hummers’ method with the modification of removing NaNO_3_ from the reaction formula, as reported elsewhere.^46^ Typically, graphite powder (1.0 g) was added to concentrated H_2_SO_4_ (25 mL) + H_3_PO_4_ (0.5 mL) under stirring in an ice bath. Under vigorous agitation, KMnO_4_ (3.0 g) was added slowly to keep the temperature of the suspension lower than 20 °C. Next, the reaction system was transferred to a 35 °C oil bath and vigorously stirred for about 0.5 h. Then, 50 mL water was added, and the solution was stirred for 15 min at 90 °C. 170 mL water was added, followed by slow addition of 5 mL H_2_O_2_ (30%) when the mixture had cooled to room temperature, turning the color of the solution from dark brown to yellow. The mixture was centrifuged and washed with 1 : 10 HCl aqueous solution to remove metal ions. A non-solvent *e.g.* diethyl ether was added for coagulation of GO. The resulting solid was dried in a vacuum oven at 60 °C for 24 h.

### Synthesis of nano-polyaniline (nPANI)

Nano-polyaniline was prepared by using polyethylene glycol (PEG-6000) as a surfactant.^47^ A solution (20 mL) containing PEG (2 g) and HCl (1 N) was mixed. Double distilled aniline (0.5 mL) was slowly added drop-wise to avoid overheating when preparing the aniline–hydrochloride solution. A freshly prepared solution of (NH_4_)_2_S_2_O_8_ (1.14 g) in 20 mL of HCl (1 N) was added drop-wise while the mixture was stirred under cool conditions. A few minutes later the suspension became green, indicating initiation of polymerization, and then the reaction was continued overnight. The resulting dark green material was filtered after washing with HCl to remove any unreacted monomer and oxidant. It was then washed several times with double distilled water and acetone followed by drying in a vacuum oven at 60 °C for 24 h. The synthesized neat polyaniline is denoted as nPANI.

### Synthesis of polyaniline nanocomposite with GO

**Fig. 1:**
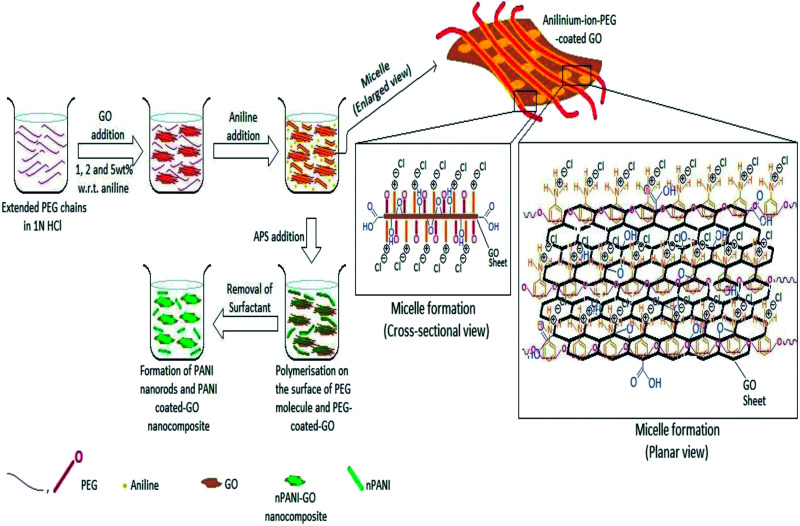
Various steps in the preparation of nPANI–GO composites. Both cross-sectional and planar views of the intermediate structures are also shown.

All the composites were prepared by *in situ* polymerization of aniline to form doped PANI. A solution (20 mL) containing PEG (2 g) and HCl (1 N) was mixed. GO was added and the mixture was kept under mechanical stirring overnight. Then, the solution was sonicated for 1 h. After sonication, double distilled aniline (0.5 mL) was added to the above solution and the mixture was mechanically stirred in an ice-bath. After that, a fresh solution containing (NH_4_)_2_S_2_O_8_ (1.14 g) in 20 mL of HCl (1 N) was added drop-wise to the solution in the ice-bath for further polymerization. The product was filtered and washed several times with distilled water. The precipitant was further washed with methanol to remove any oligomers present. Finally, the obtained dark green GO/nPANI nanocomposite powders were dried overnight at 60 °C in a traditional oven. Nanocomposites with initial loadings of 1, 2, and 5 wt% GO were synthesized and doped with HCl and are denoted as nPANI–GO 1 wt% (PGO1), nPANI–GO 2 wt% (PGO2) and nPANI–GO 5 wt% (PGO5) respectively (Fig. 1).

## Characterization

### X-ray diffraction (XRD)

**Fig. 2:**
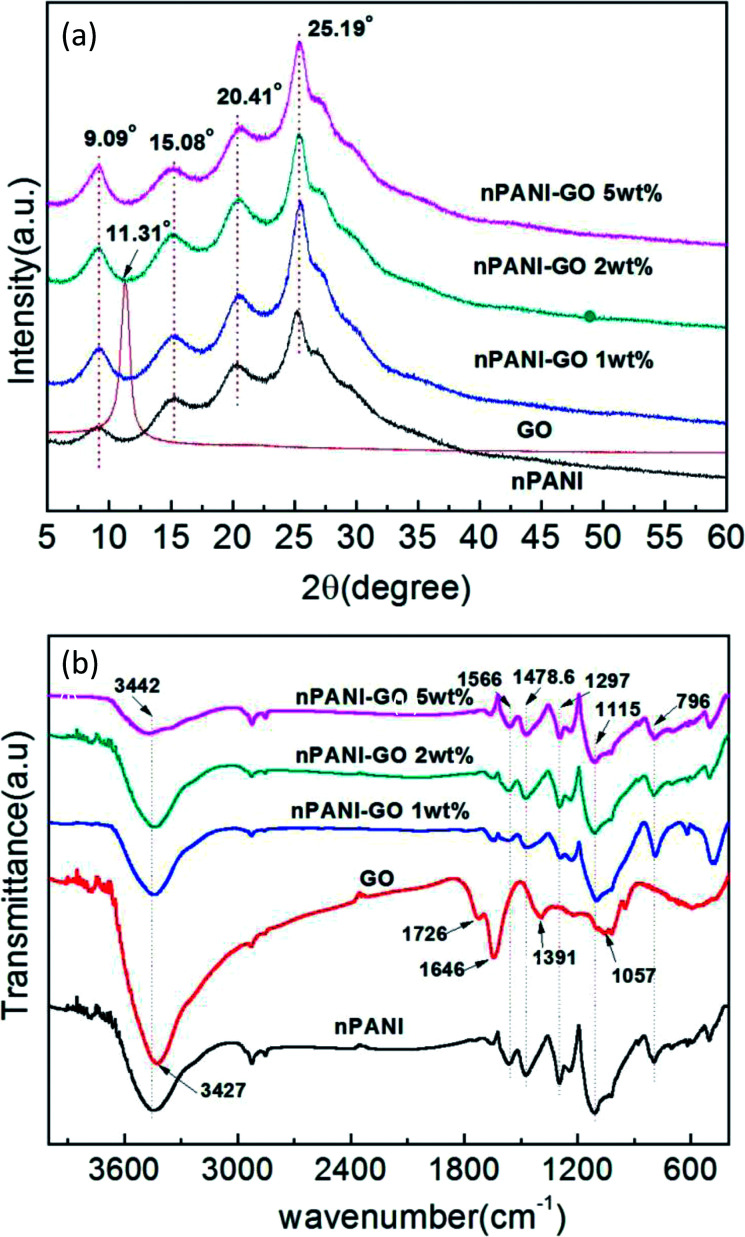
(a) X-ray diffraction patterns of GO, nPANI and nPANI–GO nanocomposites at room temperature. (b) FTIR spectra of GO, nPANI and nPANI–GO composites.

Fig. 2(a) shows the XRD patterns of GO, nano-PANI and nPANI–GO nanocomposites with varying weight percentage of graphene oxide. The diffraction data for these samples were recorded as a function of angle 2*θ* ranging from 5° to 60°. GO shows a characteristic peak at 2*θ* = 11.31° corresponding to the (001) plane, and this results in an interlayer spacing of 0.785 nm between GO sheets. It is observed that the graphite peak at 2*θ* = 26.2° cited in the literature^48^ shifts to a lower value of 2*θ* = 11.31° and this is attributed to the oxidation of pristine graphite, the intercalation of water molecules between the basal planes of the 2D surface of GO and the formation of oxygen-containing functional groups in the basal plane and at the edges of the 2D surface of the graphite crystalline structure during the process of oxidation.^49^ The peaks for nano-PANI appear at 2*θ* = 15.08°, 20.41° and 25.19° corresponding to the (011), (020) and (200) crystal planes of PANI in its emeraldine salt form.^50^Fig. 2(a) indicates that the intensity of the peak corresponding to the Miller plane (200) at 2*θ* = 25.19° is much higher than that of the other peaks, and this peak is attributed to the crystal periodicity parallel to the polyaniline chains.^39^ In the nPANI–GO nanocomposites, the GO peak disappears, which reveals that the layers of GO are completely exfoliated and well dispersed in the polyaniline matrix during the polymerization process.^17^ These peaks are assigned to crystalline regions in an amorphous matrix. The X-ray data for the nPANI–GO nanocomposites present peaks similar to those obtained for pure nano-PANI, revealing that no additional crystalline order has been introduced into the composites. However, the degree of crystallization of nano-PANI on the formation of nPANI–GO nanocomposites varies. The crystallite sizes of the PGO1, PGO2 and PGO5 samples were found to be ≈4.75 ± 0.534 nm, ≈5.30 ± 0.262 nm and ≈5.09 ± 0.128 nm, respectively.

### Fourier-transform infrared spectroscopy (FTIR)

The FTIR absorption spectra in Fig. 2(b) reveal the chemical bonding between the components in GO, nano-PANI and nPANI–GO nanocomposites with varying weight percentage of GO. For GO, the absorption bands at 1726, 1391, 1057, and 3427 cm^−1^ indicate the presence of carboxyl (–COOH), epoxy, carbonyl ( <svg xmlns="http://www.w3.org/2000/svg" version="1.0" width="10.400000pt" height="16.000000pt" viewBox="0 0 10.400000 16.000000" preserveAspectRatio="xMidYMid meet"><metadata> Created by potrace 1.16, written by Peter Selinger 2001-2019 </metadata><g transform="translate(1.000000,15.000000) scale(0.011667,-0.011667)" fill="currentColor" stroke="none"><path d="M80 1160 l0 -40 40 0 40 0 0 -40 0 -40 40 0 40 0 0 -40 0 -40 40 0 40 0 0 -40 0 -40 40 0 40 0 0 -40 0 -40 40 0 40 0 0 -40 0 -40 40 0 40 0 0 -40 0 -40 40 0 40 0 0 80 0 80 -40 0 -40 0 0 40 0 40 -40 0 -40 0 0 40 0 40 -40 0 -40 0 0 40 0 40 -40 0 -40 0 0 40 0 40 -40 0 -40 0 0 40 0 40 -80 0 -80 0 0 -40z M560 520 l0 -40 -40 0 -40 0 0 -40 0 -40 -40 0 -40 0 0 -40 0 -40 -40 0 -40 0 0 -40 0 -40 -40 0 -40 0 0 -40 0 -40 -40 0 -40 0 0 -40 0 -40 -40 0 -40 0 0 -40 0 -40 80 0 80 0 0 40 0 40 40 0 40 0 0 40 0 40 40 0 40 0 0 40 0 40 40 0 40 0 0 40 0 40 40 0 40 0 0 40 0 40 40 0 40 0 0 80 0 80 -40 0 -40 0 0 -40z"/></g></svg> C=O), and hydroxyl (–OH) groups, respectively. The peak at 1646 cm^−1^ is associated with the vibration of the absorbed water molecules and might also be due to the skeletal vibration of unoxidized graphite.^51,52^ Further, Fig. 2(b) exhibits two main peaks at 1566 and 1478.6 cm^−1^, which are attributed to C=C stretching of quinoid and benzenoid units respectively,^53^ thereby indicating the presence of doped polyaniline structures.^54^ The broad band at 3442 cm^−1^ is an indication of free charge carriers in the PANI emeraldine salt. Other absorption peaks at 1297 and 1250 cm^−1^ correspond to the C–H stretching of quinoid–benzoid–quinoid units, indicating the presence of delocalized π electrons and C–N^+^ bonds in the polaron lattice. The next bands at 1115 and 796 cm^−1^ are attributed to absorption due to the N–H stretching of the secondary aromatic amine in the quinoid–benzenoid–quinoid unit, and to in-plane aromatic C–H bending and out-of-plane C–H bending vibrations in 1,4-substituted benzene rings, respectively, indicating that the aniline units polymerized through end-to-end connections. This is a typical PANI spectrum, in accordance with those reported in the literature.^55^

### Field emission scanning electron microscopy (FE-SEM)

**Fig. 3:**
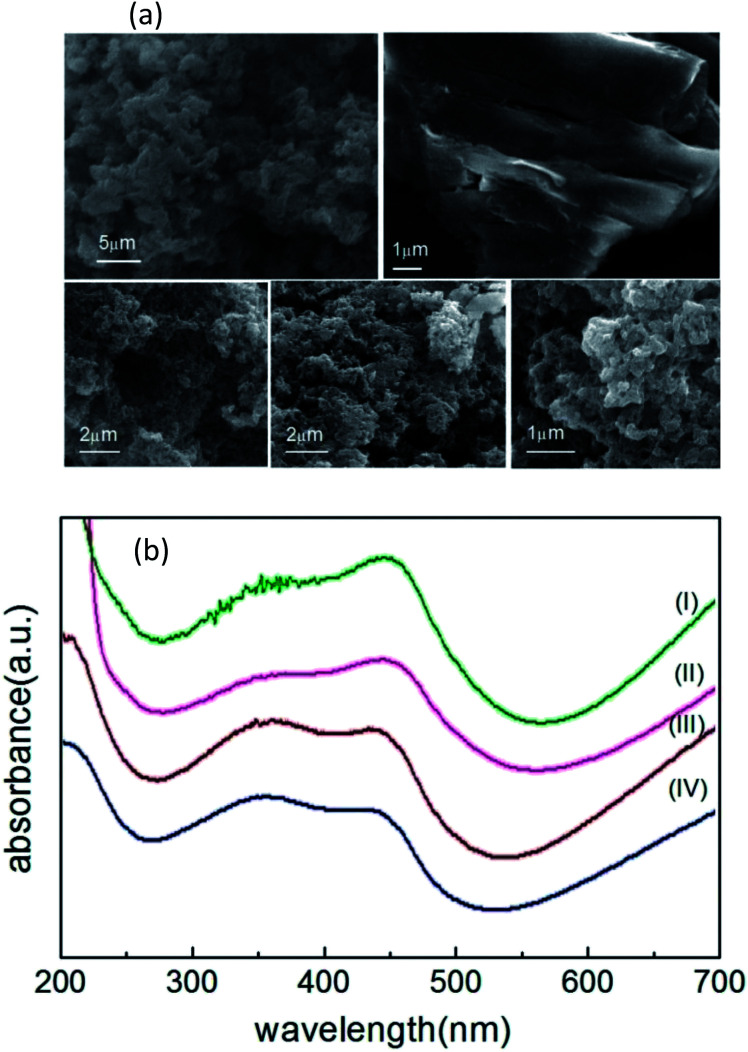
(a) Panels (I(upper left)), (II(upper right)), (III(lower left)), (IV(lower middle)) and (V(lower right)) show respectively the FESEM images of nPANI, GO, PGO1, PGO2 and PGO5. (b) The UV-visible spectra of nPANI (I), PGO5 (II), PGO2 (III) and PGO1 (IV).

Fig. 3(a) shows the FE-SEM images of carbon nanostructures, nano-PANI and nPANI–GO nanocomposites. These images reveal the dominant nanofiber morphology of PANI bonded to the surface of GO at both ends. Fig. 3a(I) shows that the nanorods of nano-PANI are tangled together. This reveals that in the presence of PEG, PANI shows a short fibrous (rod) morphology and these rods are interconnected to form a ramose structure.^47^Fig. 3a(II) shows that GO exhibits wrinkled and layered stacking up to several micrometers due to its high flexibility and opposite surface charges. Fig. 3a(III–V) show images of PGO1, PGO2 and PGO5 respectively. The wrapping capability of GO is not observed in these images due to limited space on the GO surface for the grown PANI nanofibers (*i.e.* steric hindrance), which reduces the possibility of GO wrapped PANI. It can be observed that the morphology of the nano-PANI–GO nanocomposites is significantly different from those of nano-PANI and GO, and that PANI nanofibers bridge between GO sheets. Bridging between adjacent sheets results in an interconnected network. The nPANI–GO nanocomposites exhibit multiple shapes, mainly flakes along with a short fibrillar morphology.^17^ Aniline monomers are absorbed on the surface of the GO flakes, and as the polymerization reactions proceed on the surface of the GO flakes, the resulting nanocomposites show a flaky structure.

### Ultraviolet-visible spectroscopy (UV-Vis)

Fig. 3(b) shows the UV-Vis spectra of nano-PANI and PANI nanocomposites with varying weight percentage *D* of graphene oxide GO. Within the 200–700 nm range, they (nano-PANI and PANI nanocomposites) exhibit two characteristic peaks – the absorption peak at 330–370 nm represents the π–π* transition of the benzenoid ring of nano-PANI,^56^ and the absorption peaks at 430–460 nm and 700–900 nm (not shown)^47^ can be assigned to the acid-doped state and polaron band transitions, respectively, due to protonation by HCl.^17^ In the literature, GO shows a peak at 231 nm (π–π* plasmon peak)^57^ due to sp^2^ carbon, but this peak disappears when GO is added into the PANI chain, and the nPANI–GO composites exhibit absorption peaks at 350 nm (π–π* transition) and also at 445 nm (n–π* transition, quinoid units) along with an extended tail from 600 nm (polaronic band).

### Measurement of electrical transport properties: *V*–*I* characteristics and fundamental response *V*_f_

**Fig. 4:**
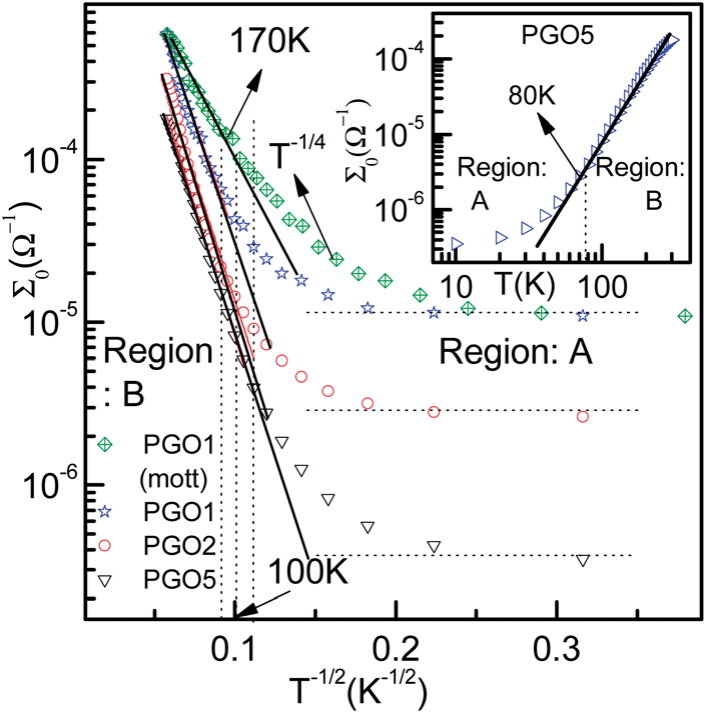
The variation of conductance *Σ*_0_ as a function of *T*^−1/2^ for PGO1, PGO2 and PGO5 samples as a semi-log plot. Solid lines are data fits obtained using eqn (2). In the same plot, the fit obtained for the PGO1 sample using Mott’s *T*^−1/4^ law (green diamonds) is also shown. Values of the fitting parameters *Σ*_p_ and *T*_0_ are shown in the third column of Table 1. The inset shows the variation of dc conductance *Σ*_0_ as a function of temperature *T* for the PGO5 sample as a log–log plot. This clearly shows two distinct regions: A and B, described in detail in the text.

**Fig. 5:**
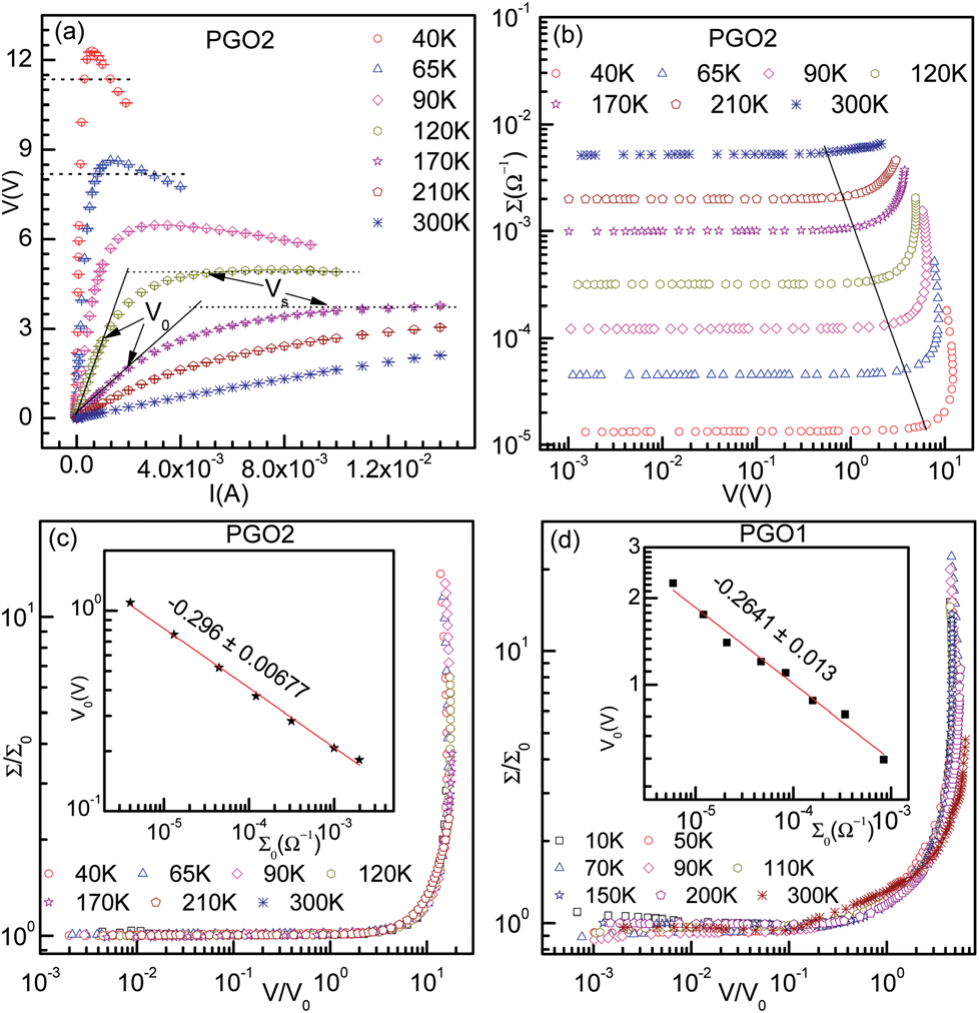
(a) The voltage–current (*V*–*I*) characteristics for the PGO2 sample at the selected temperatures shown in the graph. (b) The variation of dc conductance *Σ* = *I*/*V* as a function of *V*. This data set is obtained from the same *V*–*I* data presented in (a) and is plotted to show the onset voltages at different temperatures. (c) The scaled plot of conductance *vs.* voltage data shown in (b). The inset shows the log–log plot of onset voltage *V*_0_ as a function of ohmic conductance *Σ*_0_. The solid line is a power law fit to the data with the slope shown in the graph. (d) Similar plots for the PGO1 sample.

The samples used in this study were nanocomposites of n-PANI and GO with weight percentages of GO of 1%, 2% and 5%. These samples were in the form of thin discs of diameter 1 cm and thickness 0.3–0.4 mm. The dc (direct current) conductance *Σ* and voltage–current *V*–*I* characteristics of the samples were measured at selected temperatures by sending suitable currents from a constant current source (Keithley Model 6220) and recording the corresponding voltages across the sample with a Nanovoltmeter (Keithley Model 2182A) using the two probe method. The samples were placed in a JANIS cryostat and copper leads were connected to them using silver paste. The measurements were carried out in the temperature range 10 to 300 K, and the temperature was controlled to a precision of 0.01 K using a Lake Shore model 335 temperature controller. Data were collected by interfacing these instruments with a personal computer through the GPIB data card of a Keithley KUSB-488A converter and using LABVIEW software. Fig. 4 shows the variation of *Σ* with temperature *T* for samples containing different weight percentages of GO, and Fig. 5 shows the *V*–*I* characteristics of the sample containing 2 wt% of GO at different temperatures.

In order to understand the nonlinearity of the *V*–*I* characteristic curves, the dynamical resistance 
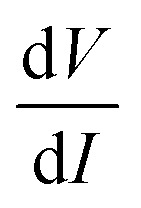
 was measured directly as a function of current *I* at selected temperatures. The measurement was performed following the standard technique of superposing a small low frequency ac modulating current on a dc bias current and detecting the fundamental response with a lock-in amplifier (LIA, Model SR830). In order to achieve this, a simple home-made circuit using an OP07 OP-AMP was fabricated, which acts as both an adder and a current to voltage converter. The dc bias current *I*_dc_ was obtained from the constant current source whereas the ac modulation *I*_AC_ sin *ωt* (*I*_AC_ = 40 nA and *f* = 33 Hz) was obtained from the internal oscillator of the lock-in amplifier. The input to the lock-in amplifier can be expressed as1
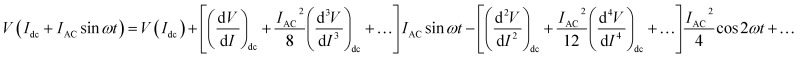


As *I*_AC_ is very small, the fundamental response is proportional to 
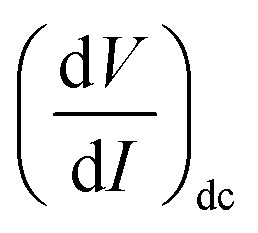
, and using the ac-coupled mode of the LIA, one can block the dc term so that one can obtain 
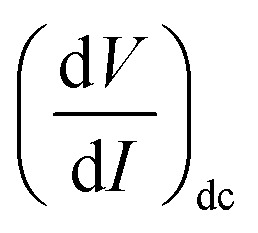
. While measuring the output signal, the time constant of the LIA was set at 100 ms. However, if the data were too noisy, especially near the saturation voltage when the signal level was very low, a larger time constant of 1 s was used in order to reduce the noise in the output signal. The constant current source and the LIA were controlled by a LABVIEW program through a GPIB. The maximum dc voltage was limited to 12 V in this work. The measurement was carried out in the temperature range from 10 to 300 K. However, one has to note that the above analysis given by eqn (1) falls through if 
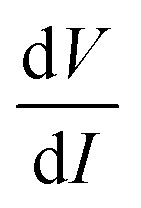
 itself is zero. In that case higher order derivatives will contribute so that it is better to write it as the fundamental response *V*_f_ instead of 
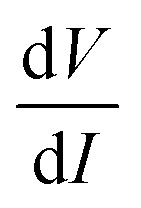
. We will discuss this issue in the Results section.

## Results and discussion

### *Σ*_0_–*T* behavior in the limit *V* → 0

The zero-voltage direct current (dc) conductance *Σ*_0_(*D*, *T*) as a function of temperature *T* of the PGO5 sample is shown as a log–log plot in the inset of Fig. 4. The result shows that *Σ*_0_ increases at a slower rate as the temperature *T* increases from a low value up to a certain temperature, say *T*_D_. With a further increase in *T* beyond *T*_D_, *Σ*_0_ increases at a greater rate, and finally at even higher temperatures, its rate of increase decreases. In order to examine the nature of the conduction mechanism in the measured temperature range, the dc conductance *Σ*(*D*, *T*) for all the PGO samples is plotted in Fig. 4 as a function of inverse square root of temperature *T* in a semi-log plot. At a fixed temperature *T*, say 50 K, it is observed that *Σ*(*D*, *T*) decreases by more than one order of magnitude with an increase in *D* from 1% to 5%. The decrease in *Σ*(*D*, *T*) with increasing *D* can be understood from the following argument: GO is composed of functionalized graphene sheets and its basal planes and edges are decorated with oxygen-containing hydrophilic functional groups such as hydroxyl, carboxyl and epoxides.^22^ These tunable functional polar oxygen-containing groups and sp^2^ hybridized carbon–carbon bonds in GO^58,59^ form the following strong interfacial interactions: (a) π–π stacking; (b) electrostatic interactions; and (c) hydrogen bonding with the nPANI polymer chains resulting in intercalated or exfoliated GO-based polymer nanocomposites.^30–32^ In addition, hydrogen bonds may be formed between the nonprotonated amine groups and hydroxyl hydrogen atoms, and also between epoxide groups and hydrogen atoms attached to electronically-charged nitrogen.^23^ These interactions are experimentally verified by UV-visible absorption, Raman, and X-ray photoelectron spectroscopy and facilitate understanding of the electrical transport properties in exploring the potential applications of these nanocomposites as supercapacitors. With the addition of a certain weight percentage *D* of GO in pristine nPANI, bonding between the nitrogen in the amine groups (nPANI) and the carbon and hydrogen in the CO and OH radicals of graphene oxide comes into effect and consequently the number of protonated sites is reduced.^18,60^ It is further revealed from confocal Raman spectroscopy that the addition of GO leads to a notable decrease in the polaron population in polyaniline, resulting in a significant increase in resistivity for the nPANI–GO-*X* nanocomposites with respect to pure polyaniline.^26^ At a fixed temperature and at a small value of *D*, this reduction in protonated polaron sites in the nPANI chain is small and an enhanced intra-chain conduction through the system is observed, resulting in a smaller value of resistance as seen in Fig. 4. With an increase in *D*, the reduction in protonated polaron amino nitrogen sites (benzenoid units) increases, thereby reducing the availability of non-bonded electrons from the nitrogen atoms. This in turn results in an increase in the number of insulating regions, and the probability of electrons hopping through the intra-chain and inter-chain networks is reduced, leading to a decrease in the conductance of the nanocomposite as shown in Fig. 4.

At a fixed wt% *D* of GO, say for the PGO1 sample, conductance *Σ*(*D*, *T*) remains almost constant at low temperature (>10 K) when plotted as a function of *T*^−1/2^. This is shown by the dotted line in Fig. 4. With an increase in *T*, *Σ*(*D*, *T*) increases very slowly from this (almost) constant level. With a further increase in *T*, *Σ*(*D*, *T*) increases at a faster rate and by almost one and a half orders of magnitude in the measured range of temperature. Above a certain temperature *T*_D_ (for PGO1, *T*_D_ = 120 K), *Σ*(*D*, *T*) increases very rapidly. Similar results were also observed for both the PGO2 and PGO5 samples, for which the increase in conductance within the same temperature range was more than three orders of magnitude. The values of *T*_D_ for these two samples were found to be 100 K and 80 K, respectively, which indicates an increase in the value of *T*_D_ with a decrease in *D*. The region within the temperature range from room temperature down to *T*_D_ is marked as “region B”. Below *T*_D_, *Σ*(*D*, *T*) decreases at a slower rate with decreasing *T*. The region within *T*_D_ ≥ *T* ≥ 10 K is termed as “region A”. Thus, *T*_D_ marks a transition from a weakly insulating region (region A) at low temperatures to a region that obeys Mott’s law of variable range hopping with the temperature exponent *γ* = 1/2 at higher temperatures (region B). The *Σ*(*D*, *T*)–*T* data for all the PGO samples in region B were fitted with the variable range hopping formula modified for the Coulomb interactions as mentioned below:2
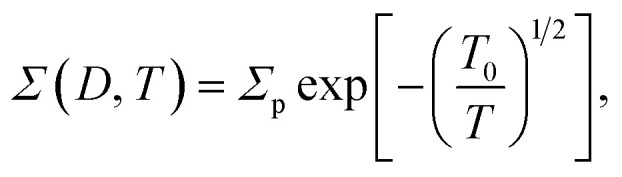
where *Σ*_p_ is the conductance prefactor and *T*_0_ is the characteristic fitting parameter linked to the apparent activation energy. Values of *Σ*_p_ and *T*_0_ for all the samples are shown in column III of Table 1. The excellent fits obtained with eqn (2) imply that the aforementioned dependence of *Σ*(*D*, *T*) on *T* is dominant in region B (*T* > *T*_D_). This is because of the electron–electron Coulomb interaction at the Fermi level. This interaction opens up a soft gap at the Fermi level, reducing the density of states at the Fermi level.^61^ This is evidence for a phonon-assisted tunneling mechanism for charge carrier transfer among the conductive grains of the nanocomposites^25^ and results in the dependence of conductance on *T* as given by eqn (2). The conductance in region A (10 K ≤ *T* ≤ *T*_D_) is still dominated by phonon-assisted hopping between localized states, which competes with the extended electronic transport. Eqn (2) has also been applied to nPANI protonated by exposure to HCl, suggesting three types of model for temperature dependence, such as quasi-1D variable range hopping between the nearest-neighbour chains, hopping in granular metals, and 3D variable range hopping in the presence of a Coulomb gap at the Fermi level, and has been used by several authors to interpret the temperature dependence data for nPANI conducting polymers and composites.^13,17,25,40,41,62^ Recently, Neti *et al.*^25^ observed this type of transition at *T*_D_ = 75 K for a conducting polyaniline/graphene oxide composite and concluded that the transition is influenced by factors like temperature and annealing process. In order to make a comparison, the *Σ*_0_–*T* data (shown by the green diamond symbol in Fig. 4) for the PGO1 sample were fitted with Mott’s *T*^−1/4^ law, which exhibited a good fit up to 170 K, but the fitting with eqn (2) was better up to 120 K and was found to be consistent with the change in value of the onset exponent for alternating current conduction.^63^ To show the data in the same temperature range, a factor of −0.18258 has been added to the *T*^−1/4^ data. Further, the electrical transport mechanism in these nPANI–GO nanocomposites is explained by Arrhenius behavior at high temperatures (*T* > 255 K) and by a broad transition with a logarithmic dependence of the activation energy on temperature for the low temperature regime (*T* < 255 K).^26^ At intermediate temperatures, the Arrhenius plots usually show an apparent deviation from linearity described by the Larkin and Khmel'nitskii prediction of a logarithmic dependence of the activation energy for disordered systems with large localization length.^64^ However in our sample, the *T*-dependence of ohmic conductance *Σ*_0_ is successfully described by eqn (2) below *T*_D_.

**Table 1:** Structural parameters as calculated from room temperature XRD and *R*–*T* data for the nPANI–GO-*D* nanocomposites

Systems studied	Size from XRD (nm)	Fitting parameters		*x*_T_
		*Σ*_p_ (Ω^−1^)	*T*_0_(*T*)	
nPANI–GO (*X* = 1%)	≈4.75 ± 0.534	3.70 × 10^−3^	799.2	−0.264 ± 0.013
nPANI–GO (*X* = 2%)	≈5.30 ± 0.262	2.70 × 10^−3^	912.04	−0.296 ± 0.0067
nPANI–GO (*X* = 5%)	≈5.09 ± 0.128	2.48 × 10^−3^	1056.25	−0.318 ± 0.0126
r-GO^44^				−0.72 ± 0.20
PANI–ClAlPc^45^				−0.07 ± 0.0132
Bulk polypyrrole^65^				−0.329 ± 0.014
Polypyrrole film^65^				−0.155 ± 0.012
nPANI/pSi^43^	30–50			−0.30 ± 0.08

It should be mentioned here that the increase in resistance of the nanocomposite with increasing *D* can be explained using knowledge of the characteristic temperature *T*_0_ in the following way: fits to *Σ*(*D*, *T*)–*T* data with eqn (2) show that *T*_0_ increases with *D* (see Table 1), and this is commonly associated with reduction of the conductive protonated phase and shrinking of the conducting grains in nPANI.^25^ The parameter *T*_0_ is directly related to the ratio of the average distance between neighbouring conductive grains *d* and the average grain size *s* by the following relation:^25^3
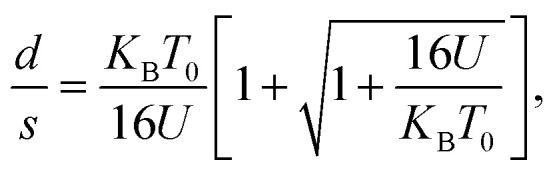
where *U* is the Coulomb repulsive energy between two electrons at a distance equal to the size of an nPANI monomer and *K*_B_ is the Boltzmann constant. Further, it is assumed that *U* retains a constant value as a function of *D* (in the limit of low *D*) and is roughly equal to 2 eV.^25^ The ratio 
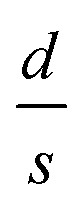
 for the three samples was calculated using eqn (3) and was found to be 0.0486, 0.0525 and 0.0555 for PGO1, PGO2 and PGO5, respectively. This remarkable increase in the 
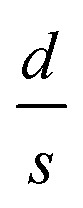
 ratio reflects the striking reduction in the grain size *s* while the separating barrier width *d* follows the opposite trend. This simple calculation shows the resultant broadening of the insulating regions at the expense of shrinking of conducting grains and accounts for substantial disorder which disturbs the extended conduction network and causes the electronic states to become localized. This localization of the electronic states increases with an increase in *D* and results in an increase in the resistance of the nanocomposites with increasing *D* as reflected in Fig. 4.

### *V*–*I* characteristics

The typical *V*–*I* characteristic curves of the PGO2 sample at selected temperatures are shown in Fig. 5(a). Depending upon the range of current values, these curves exhibit various interesting features. At a particular temperature, say 300 K, the voltage *V* across the sample increases linearly with the applied current *I*, exhibiting the ohmic character of the *V*–*I* curves. With a further increase in *I*, *V* increases at a slower rate, *i.e.* the *V*–*I* curve deviates from linearity. The value of current *I* at which the *V*–*I* curve at a particular *T* deviates from linearity is defined as the onset current *I*_0_(*T*) and the corresponding voltage is termed the onset voltage *V*_0_(*T*). The position of *V*_0_(*T*) is marked on the two *V*–*I* curves corresponding to 120 K and 170 K in Fig. 5(a). With a further increase in *I* beyond *I*_0_(*T*), *V* approaches a saturation value *V*_S_(*T*) which is maintained over the measured current range. *V*_S_(*T*) is shown on the same two *V*–*I* characteristic curves corresponding to the temperatures *T* = 120 K and 170 K. This saturation in voltage at *V*_S_(*T*) is observed only in the *V*–*I* curves measured above temperature *T*_D_. *V*_S_(*T*) increases with decreasing temperature, and saturation occurs at lower values of current. Another interesting feature is observed in the *V*–*I* characteristic curves measured at temperatures below *T*_D_. Beyond the onset voltage *V*_0_(*T*), voltage *V* reaches a maximum (say, *V*_max_) at a certain value of current (say, *I*_max_) and then decreases with an increase in current *I*. This decrease in *V* is not due to Joule heating and a detailed discussion will be presented in the section on “Saturation of voltage: Zener-like behavior”. Similar features in the *V*–*I* characteristic curves were observed for the PGO1 and PGO5 samples.

The inherent variations in the evolution of *V*–*I* curves shown in Fig. 5(a) can be better illustrated by plotting the *V*–*I* curves as *Σ*(*T*, *V*)–*V* curves as shown in Fig. 5(b). The symbol *Σ*(*T*, *V*) refers to the fact that the conductance of the sample with a fixed wt% *D* of graphene oxide varies with temperature *T* and is probed as a function of voltage *V*. The general feature of these curves is that at a fixed temperature *T*, the conductance *Σ*(*T*, *V*) remains almost constant at its zero voltage ohmic value *Σ*_0_(*T*) up to the onset voltage *V*_0_(*T*). With a further increase in *V* beyond *V*_0_(*T*), *Σ*(*T*, *V*) increases at a greater rate, exhibiting its non-ohmic character. *V*_0_(*T*) thus marks the transition from an ohmic (linear) to a non-ohmic (nonlinear) regime and is a function of temperature. Both *V*_0_(*T*) and *V*_S_(*T*) decrease with an increase in *T*, but *Σ*_0_(*T*) increases with *T* (see Fig. 4). It is intriguing that the variations present in the *Σ*(*T*, *V*)–*V* curves at different temperatures can be traced out by tracking the dynamics of the onset voltage *V*_0_(*T*) as a function of temperature *T* as shown by the solid line in Fig. 5(b). The orientation of this line clearly indicates that *V*_0_(*T*) is closely related to the temperature *T* and the zero-voltage conductance *Σ*_0_(*T*). This experimental result provides an effective way of electrical characterization of non-ohmic conduction in these nanocomposites and explores the possibility of their practical applications in technological fields.

Fig. 5(c) shows the result of merging the *Σ*(*T*, *V*)–*V* data presented in Fig. 5(b) into a master curve following the data collapse method.^65,66^ This method is described in detail below: the conductance *Σ*(*T*, *V*) corresponding to the curve at a certain temperature, say *T* = 40 K, is divided by its ohmic value *Σ*_0_(*T*) and the voltage of the same curve is kept unaltered, *i.e.* the onset voltage *V*_0_(*T*) corresponding to this curve is chosen to be 1. For the next higher temperature (*T* = 65 K), the conductance *Σ*(*T*, *V*) is also divided by *Σ*_0_(*T*) as before, but *V*_0_(*T*) is adjusted in such a way that the *Σ*(*T*, *V*)–*V* curve merges with the previous one as well as possible. This procedure is then repeated for the other *Σ*(*T*, *V*)–*V* curves corresponding to different temperatures. The master curve is shown in Fig. 5(c), which clearly exhibits the signature of excellent data collapse up to *Σ*(*T*, *V*)/*Σ*_0_(*T*) ≈ 15 and proves the existence of a voltage scale *V*_0_(*T*) for non-ohmic conduction in these nanocomposites at different temperatures. In the inset of Fig. 5(c), *V*_0_(*T*) is plotted against *Σ*_0_(*T*) on log–log axes. The solid line is a fit to the data obtained using the following phenomenological relation:^65,66^

4 *V*_0_(*T*) = *A*_T_*Σ*_0_(*T*)^*x*_T_^

where *A*_T_ is a constant and *x*_T_ is the onset exponent. The constant *A*_T_ is determined by the criterion that fixes the voltage scale *V*_0_(*T*) at temperature *T* and the onset exponent *x*_T_ determines the level of nonlinearity in the system. The fit to the *V*_0_(*T*) *vs. Σ*_0_(*T*) data obtained using eqn (4) yields a value of *x*_T_ of −0.296 ± 0.007. The data collapse method was also used to achieve master curves for the PGO1 and PGO5 samples. A scaled 
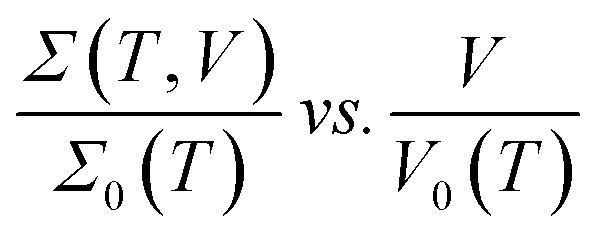
 curve for the PGO1 sample is shown in Fig. 5(d) and the nice collapse of the scaled data indicates that this scaling formalism is also valid for achieving the master curve for this sample. In the inset of Fig. 5(d), *V*_0_(*T*) for this sample is shown as a function of *Σ*_0_(*T*) on log–log axes, and the fit obtained with eqn (4) provides a value for the nonlinearity exponent *x*_T_ = −0.264 ± 0.013. This *x*_T_ is slightly larger than the value for PGO2. Following the same procedure, the value of *x*_T_ for PGO5 was found to be −0.318 ± 0.0126. All these values of *x*_T_ are shown in the fourth column of Table 1.

### Fundamental response *V*_f_

**Fig. 6:**
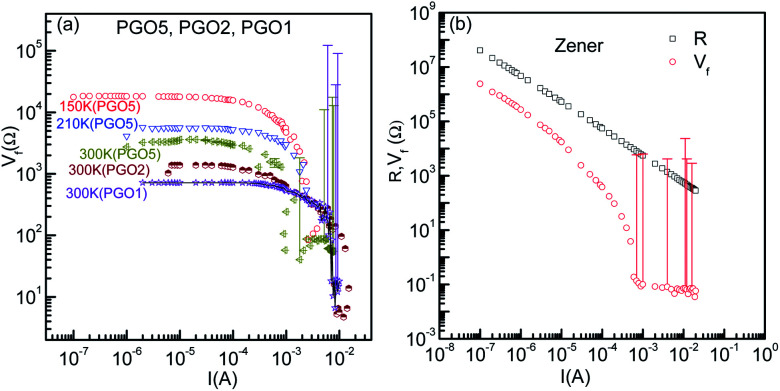
(a) The variation of fundamental response *V*_f_ as a function of current *I* for the PGO1, PGO2 and PGO5 samples at the selected temperatures mentioned in the graph. (b) Similar plots for a commercial Zener diode together with the variation of chordal resistance *R* with *I* in the breakdown region. Error bars for the fundamental responses are indicated in both figures.

In order to investigate further the nonlinear features of the *V*–*I* characteristics, the fundamental response *V*_f_ of the PGO samples was investigated as a function of current *I* in the ohmic, non-ohmic and saturation regions, and the results of these measurements are shown in Fig. 6(a). The samples and the temperatures at which the fundamental response *V*_f_ was measured are mentioned next to each curve. These results reveal that *V*_f_ has qualitatively similar characteristic features at different temperatures. For example, the fundamental response *V*_f_ for the PGO5 sample at a temperature of 150 K (>*T*_D_) remains constant at ∼20 kΩ up to the onset current *I*_0_ (in the linear region) and decreases steadily towards a very small value of ∼30 Ω as the applied current increases above *I*_0_. Such a small value of *V*_f_ at higher current (*I* ≥ *I*_0_) is consistent with the saturation of the *V*–*I* characteristics at a particular temperature. Similar results were also observed for the PGO1 and PGO2 samples at various temperatures above *T*_D_. To compare the voltage saturation at higher currents with that of a Zener diode, the fundamental response *V*_f_ of a commercial Zener diode (1N4734) was measured as a function of current *I* in the breakdown region using a home made circuit, and the results of this measurement are shown in Fig. 6(b) (open circles). In the same figure, the corresponding resistance (open squares) is also shown as a function of current *I*. In this case, the fundamental response *V*_f_ decreases from ∼2.4 × 10^6^ Ω at lower currents to ∼5 × 10^−2^ Ω at higher values of current, whereas the resistance *R* decreases from ∼4 × 10^7^ Ω to ∼2.8 × 10^2^ Ω in the same range of applied current. This small value of *V*_f_ at higher currents for the Zener diode signifies true saturation of voltage and also shows that the home made circuit functions properly in the measurement of the fundamental response *V*_f_.

The results obtained are discussed in detail in this section. But before presenting this discussion, the structural details of the nPANI–GO composites are described fully, as they are closely related to the experimental results for electrical conduction. From the X-ray diffraction studies it is clear that the polyaniline loaded on graphene oxide is crystalline. nPANI–GO thus may be regarded as a heterogeneous system composed of partially ordered crystalline regions and disordered amorphous regions upon doping.^67^ The crystalline domain is metallic in nature (called metallic islands). The conduction in this region occurs through electron delocalization or hopping of the charge carriers after the formation of polarons.^68^ The metallic domain is surrounded by the amorphous region which contains disordered or folded chains. Tunneling or hopping of the charge carriers between two metallic islands can occur through these amorphous regions, and the charge transport is governed by the disorder present. The disorder in the conducting polymer systems can arise from variations in the conjugation length, rotations and kinking of the polymer chains, van der Waals interactions with neighboring conjugated molecules, impurities, dipole moments of the neighboring dopant molecules and dipole moments of the molecules of the polymer matrix. These crystalline polyaniline chains are loaded on the less conducting amorphous graphene oxide matrix. This physical picture of the nPANI–GO composites will be utilized for the explanation of the non-ohmic electrical conduction as a function of voltage and temperature.

The typical *V*–*I* characteristics of the samples of nPANI–GO nanocomposites shown in Fig. 5 exhibit four distinct regions: (i) at high temperatures and at small currents, the *V*–*I* curves exhibit linear characteristics up to *V*_0_(*D*, *T*) (linear region); (ii) the *V*–*I* curves deviate from linearity with increasing current, and in this case, the conductance *Σ* increases from its ohmic value *Σ*_0_(*T*) (onset of nonlinear conduction at *V*_0_(*D*, *T*)); (iii) with a further increase in current (∼ mA), the voltage *V* approaches the saturation value *V*_S_ and the fundamental response *V*_f_ → 0 (saturation region above *V*_S_) which occurs at a lower value of current with decreasing temperature for a sample with fixed wt% of GO; and (iv) with a further decrease in temperature below *T*_D_, the *V*–*I* characteristics go through a maximum and the fundamental response *V*_f_ becomes negative (negative differential resistance). The maximum becomes sharper with decreasing temperature. Similar types of *V*–*I* characteristics were observed for all the PGO samples studied. In this article, regions (i), (ii) and (iii) are described in detail with less emphasis on region (iv).

### Linear region

The results presented in Fig. 5(a) and (b) and 6(a) indicate the existence of a linear (ohmic) region in the *V*–*I* characteristics. These linear characteristics can be understood from the above mentioned structural features of the nPANI–GO nanocomposites. When the emeraldine base is doped in an acidic environment, self-localized electron states called polarons or bipolarons are induced through successive formations of positive species. A polaron has a spin of 
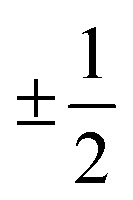
 and a charge of ±*e* whereas bipolarons are spinless with charge ±2*e*. Bipolaron structures are thermodynamically more stable and conductive.^69^ These polarons or bipolarons play a vital role in controlling the conduction mechanism in conducting polymers such as polyaniline.^39,68^ A polaron is an electronic carrier self-trapped in a potential well produced by the deformation of the molecule it occupies. Its energy is determined by electron–phonon coupling through Coulomb interactions. In polaron structures, a cationic radical on one nitrogen acts as a hole, which can transfer an elemental positive charge. The electron from the adjacent nitrogen (neutral) jumps to this hole, and it becomes electrically neutral, initiating motion of the holes through a resonance process.^39,68^ This results in electron transportation along the chain in polyaniline and, thus, p-type electrical conductivity is achieved.^69^ When the applied voltage (or current) is small, conduction is mainly dominated by the nPANI chains through the hopping of charge carriers resulting in the ohmic behaviour of the *V*–*I* characteristics which continues up to the onset voltage *V*_0_. The major contribution to ohmic conductance originates from the intra-chain hopping conduction through the nPANI chains.

### Onset of nonlinear conduction

It is evident from Fig. 5(b) that the conductance *Σ*(*T*, *V*) increases from its ohmic value *Σ*_0_(*T*) at the onset voltage *V*_0_. The orientation of the solid line in the figure indicates that with an increase in temperature *T*, *Σ*_0_(*T*) increases but *V*_0_ decreases, making the onset exponent *x*_T_ negative (defined in eqn (4)). Further, the absolute value of *x*_T_ increases with an increase in weight percentage of GO. These observations can be physically understood from the following picture of the nanocomposites. It is argued that the partially protonated regions in the EB form of polyaniline can be readily considered as conducting grains separated by unprotonated polyaniline.^40^ Fully protonated polyaniline, amorphous polyaniline, nonstoichiometry and contacts between polyaniline chains are regarded as insulators separating these conducting grains. The charge carriers move through these conducting grains and also hop through the insulating regions, contributing to conductance influenced by voltage and temperature. The length of such a hop *L*_hop_ is equal to the localization length *L*_c_ at the transition temperature *T* ∼ 80 K for the PGO5 sample and increases in relation to *L*_c_ with decreasing temperature.^26^ For a sample with fixed *D*, *L*_hop_ increases with a decrease in temperature and the neighbouring nPANI conducting grains become accessible to the charge carriers. As the applied voltage is increased, the number of polarons and bipolarons formed increases rapidly and neighboring nPANI conducting grains start to connect to each other through tunneling of charge carriers.^42^ This results in an increase in conductance above its ohmic value at a voltage known as the onset voltage *V*_0_. As the temperature increases, *L*_hop_ decreases, leading to lower accessibility of nPANI neighboring grains. This requires a smaller voltage to start the onset of non-ohmic conduction, making *V*_0_ smaller. This in turn makes the onset exponent *x*_T_ negative. Further, it is observed that the absolute value of *x*_T_ increases with *D*. This can be understood from the following physical explanation. At a fixed temperature *T*, the microstructure of the nPANI–GO nanocomposite is set at a fixed concentration *D* of GO and the nonlinearity in the current–voltage characteristics occurs at a constant voltage *V*_0_. Further, at a fixed *T*, the conductance decreases with an increase in *D*. As a result, eqn (4) demands that *x*_T_ has to increase in order to achieve the onset voltage *V*_0_(*T*), causing nonlinearity in the *V*–*I* characteristics. This is observed experimentally.

Negative values of *x*_T_ have been reported by several authors for various forms of conducting polymers such as bulk samples of FeCl_3_ doped polypyrrole^65^ and thin films of polypyrrole.^65^ We digitized the *V*–*I* data for different polymer systems/composites like reduced graphene oxide,^44^ PANI–ClAlPc nanocomposites^45^ and heterojunctions of nano-PANI/porous silicon^43^ and scaled them using the data collapse method to obtain the values of *x*_T_. In all these systems, *x*_T_ was found to be negative, and these values are shown in the fourth column of Table 1. In most of these studies, the aforementioned arguments have also been invoked to explain the onset of non-ohmic conduction and the negative value of the nonlinearity exponent *x*_T_.

### Saturation of voltage: Zener-like behavior

**Fig. 7:**
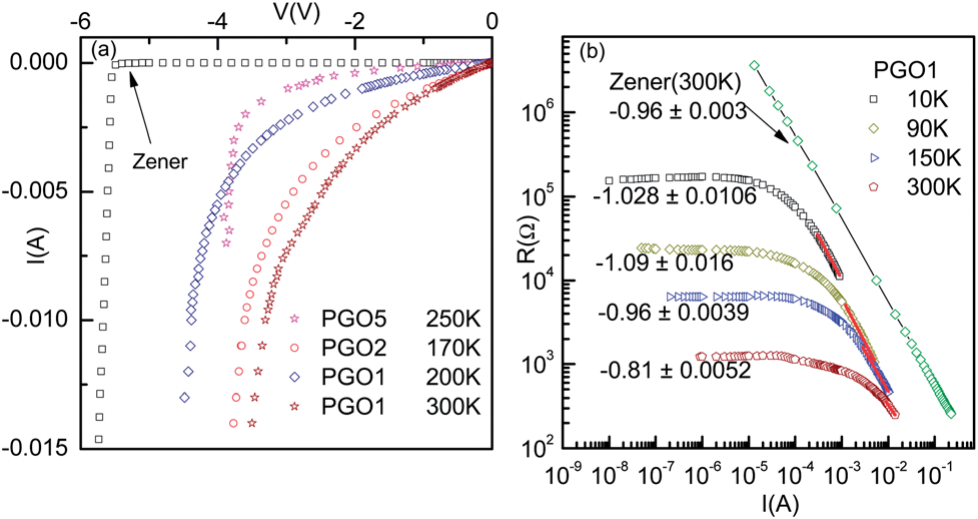
(a) Current–voltage characteristics (in the third quadrant) of the PGO1, PGO2 and PGO5 samples at the selected temperatures mentioned in the figure. For comparison, the current–voltage characteristics of a commercial Zener diode are also shown. (b) Resistance–current plot for the PGO1 sample. Data corresponding to the nonlinear regions were fitted with a power law and the corresponding exponents are marked against each curve. The values of the exponents were found to be close to unity. A similar plot of the data corresponding to the breakdown region of the Zener diode and the exponent are also shown for comparison.

It is evident from the experimental results recorded above *T*_D_ and presented in Fig. 5(a) (see the curve at *T* = 170 K) that at higher current, the voltage becomes saturated beyond *V* > *V*_S_. Further, the variation in the fundamental response *V*_f_ as a function of *I* shown in Fig. 6(a) supports the saturation of voltage. This saturation of voltage at higher currents is shown in Fig. 7(a) for all the nanocomposites at selected temperatures together with that for a commercial Zener diode at room temperature. Following the conventional procedure, the *I*–*V* curves were plotted in the third quadrant. In the case of the Zener diode, the current increases sharply at the breakdown voltage as usual. Similar behavior was also observed for the PGO samples at different temperatures. It should be mentioned here that the PGO samples do not possess the rectifying property which is an inherent property of a Zener diode. Further, the saturation voltage *V*_S_ was found to decrease with an increase in *T* as can be seen in Fig. 7(a) for the PGO1 sample at temperatures 200 K (*V*_S_ ∼ 4.2 V) and 300 K (*V*_S_ ∼ 2.8 V).

As the voltage across the Zener diode is constant in the breakdown region, its resistance can be written as

5 *R* = *A*_R_*I*^−1^.

**Fig. 8:**
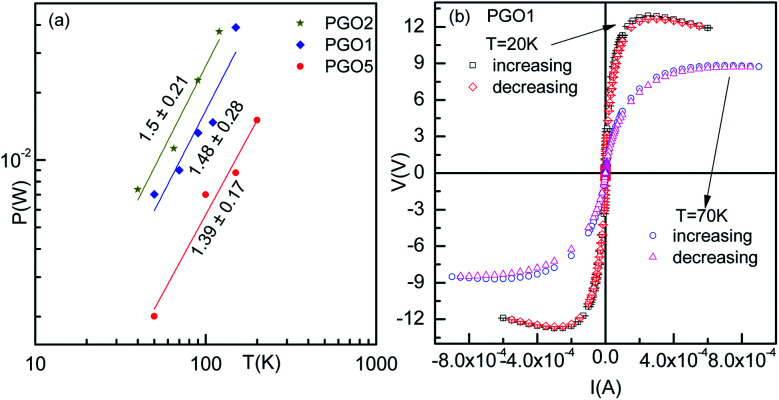
(a) Power–temperature characteristics of PGO samples corresponding to the maximum voltage at different temperatures. (b) The voltage–current characteristics of the PGO1 sample with increasing and decreasing voltages at two selected temperatures, 20 K and 70 K.

Here *A*_R_ is a constant depending upon the temperature and disorder. Eqn (5) indicates that a log–log plot of *R* as a function of *I* would provide a slope *x*_Z_ = −1. This is indeed seen (black line) in Fig. 7(b) for a Zener diode with *x*_Z_ = −0.96 ± 0.003. Following a similar approach, the *R*–*I* data for the PGO1 sample at different temperatures were fitted with eqn (5) (red lines) at higher currents. The values of *x*_Z_ are indicated against each curve. The fits are equally good, confirming the Zener-like behavior of the nanocomposites above temperature *T*_D_. Below *T*_D_, the *V*–*I* characteristics of the PGO samples exhibit a maximum voltage *V*_max_ at a certain value of current (say, *I*_max_), and with a further increase in current beyond *I*_max_, the voltage decreases. *I*_max_ shifts towards a lower value with a decrease in *T*. To find the relation between the power *P* corresponding to this *V*_max_ and the temperature *T*, *P* was plotted against *T* in a log–log plot as shown in Fig. 8(a). This shows a power law behaviour given by *P* ∼ *T*^*p*_T_^ where *p*_T_ is the corresponding exponent with a value nearly equal to 1.50. This feature is consistent with an increase in resistance with a decrease in *T* and needs theoretical explanations.

Fig. 5(a) shows the *V*–*I* characteristics of the PGO2 sample at selected temperatures. The two dotted lines drawn on the *V*–*I* curves corresponding to temperatures 40 K and 65 K indicate that a fixed value of voltage corresponds to two values of current. This shows a negative differential resistance. In order to check whether the negative differential resistance was a result of heating, the *V*–*I* characteristics were measured for both increasing and decreasing values of current and also on reversing the current. Our aim was to see whether any hysteresis was present which might be due to heating. But the experimental results shown in Fig. 8(b) indicated no hysteresis in the data, confirming that heating has almost no effect on the phenomenon. In the case of the *V*–*I* data, the negative differential resistance (NDR) phenomenon is nothing new. However, in the literature, it has been shown that the current decreases with increasing voltage. But in our case, the voltage decreases with increasing current. These two situations are not exactly the same. In fact, in our case the situation is the same as that found in thyristors, very well-known devices used to control power and voltage. This situation signifies the possible application of this nanocomposite as a thyristor.

## Conclusions

In summary, the synthesis, characterization and electrical transport properties of nPANI–GO nanocomposites with GO content ranging from 1 to 5 wt% have been explored. The nanocomposites were prepared by the *in situ* polymerization route using polyethylene glycol (PEG) as a non-ionic surfactant to achieve better dispersion and formation of nano-PANI (nPANI). The FE-SEM results showed that the GO was well covered with a PANI matrix. The XRD results indicated high crystallinity in the nanocomposites due to doping with DBSA during the polymerization process. Interactions between GO and PANI to form nanocomposites were confirmed by the UV-Vis and FTIR results. Over the measured temperature range, the nanocomposites exhibited two phases: insulating in the temperature range from room temperature down to temperature *T*_D_, and a weakly temperature dependent behavior below *T*_D_ down to 10 K. *T*_D_ was found to vary with weight percentage of GO. The *V*–*I* characteristics of the nPANI–GO nanocomposites exhibited non-ohmic electrical conduction characterized by the onset voltage *V*_0_ which scales with ohmic conductance *Σ*_0_ with the nonlinearity exponent *x*_T_. *x*_T_ has negative values that vary with the weight percentage of graphene oxide. In the temperature range from room temperature to *T*_D_, the nanocomposites show Zener-like behavior with the saturation voltage *V*_S_ increasing with decrease in temperature. Below *T*_D_, they show thyristor-like behavior. *V*_max_ decreases with decrease in *T*. These results were explained by considering the motion of the charge carriers such as polarons and bipolarons in the physical structure of the nanocomposites, which consists of conducting grains separated by insulating regions.

## Conflicts of interest

There are no conflicts to declare.

## Supplementary Material
